# Odorless Glutathione Microneedle Patches for Skin Whitening

**DOI:** 10.3390/pharmaceutics12020100

**Published:** 2020-01-27

**Authors:** Yechan Lee, Sujeet Kumar, Sou Hyun Kim, Keum-Yong Seong, Hyeseon Lee, Chaerin Kim, Young-Suk Jung, Seung Yun Yang

**Affiliations:** 1 Department of Biomaterials Science, Life and Industry Convergence Institute, Pusan National University, Miryang 50463, Korea, , , , kimchaerin12@naver.com (C.K.); 2 College of Pharmacy, Pusan National University, Busan 46241, Korea; hyunie1728@naver.com

**Keywords:** glutathione, transdermal drug delivery, hyaluronic acid, microneedle, skin whitening

## Abstract

Glutathione is a natural anti-aging substance that prevents the oxidation of protein thiols from reactive oxygen species. In the pharmaceutical industry, reduced glutathione (GSH) has been widely used for skin whitening due to its ability to inhibit tyrosinase. However, its poor permeability and foul odor limit its use in skin applications. Herein, we report a GSH-loaded dissolving microneedle (MN) patch prepared with hyaluronic acid (HA) that enables enhanced permeation across the skin and reduces the foul odor of GSH. HA was selected to prepare odorless GSH solutions and used for MN fabrications as a carrier of GSH. GSH-loaded MN (GSH-MN) arrays prepared from MN-forming solution containing up to 10% GSH showed good pattern uniformity and appropriate mechanical properties for insertion into the skin. The GSH-MNs with a loading capacity of 17.4% dissolve within 10 min following insertion into porcine skin and release the loaded GSH without being oxidized. This new approach combines functional biopolymers to reduce the characteristic GSH odor and advanced transdermal delivery based on MN technology to enhance skin permeation without pain. We believe this technique could expand the application of GSH in many cosmeceutical fields.

## 1. Introduction

Glutathione, a naturally occurring thiol tripeptide of γ-glutamyl-cysteinyl-glycine, plays a vital role in cellular redox reactions and is involved in the inhibition of melanin synthesis, protection from reactive oxygen species, and cell detoxification [1,2]. While glutathione is present in both oxidized and reduced forms within the body, reduced glutathione (GSH) is a compound with a high degree of biological utility [3,4]. For instance, its ability to suppress melanin synthesis through tyrosinase inhibition allows it to be used as a skin whitening agent in cosmeceuticals [5,6,7]. In addition, GSH may be used as an immune booster, an antidote for metal poisoning, and for the treatment of diseases such as fibrosis, glaucoma, and arthritis [8,9,10,11]. Unfortunately, its poor bioavailability and unpleasant odor limit the use of GSH in clinics despite its many therapeutic applications.

Medicinal peptides are commonly administered by parenteral routes using hypodermic needles. However, this route requires medical attention for each dose administration and has poor patient compliance due to the pain experienced during injection [12,13]. While topical applications have been used to deliver GSH across the skin, its foul odor and inability to pass through the stratum corneum (SC, the outermost barrier of skin) limit its use [14].

Recently, a minimally invasive system of transdermal delivery using microneedles (MNs) has received great attention from medical practitioners, particularly for the delivery of bioactive agents such as peptides and proteins that are labile to gastrointestinal pH and susceptible to enzymatic degradation [15].

To overcome the limitations associated with existing methods of GSH delivery, we envisioned a fast-dissolving MN patch prepared by deodorizing biopolymers to improve the efficacy of GSH and patient compliance. The high biocompatibility and tunable physicochemical properties of biopolymers such as hyaluronic acid (HA) render them suitable candidates as a MN material [16,17]. In addition, biopolymers with multifunctional groups can interact with molecules through reversible ionic interactions, hydrogen bonding, and van der Waal forces, thereby contributing to their high loading capacity and good adsorbent properties [18]. Since the release kinetics of drugs loaded in MNs may be controlled through dissolution or the degradation rate of the polymers used, dissolving MN platforms can be applied for the sustained and long-term release of drugs through transdermal routes [19,20,21,22].

Here, we report a new approach for the efficient and odorless transdermal delivery of GSH using dissolving MN patches prepared from deodorizable biopolymers. After screening several biopolymers for odorless GSH formulations, HA was selected as a material for the MN and used for the fabrication of GSH-loaded MN patches. The quantity of GSH loaded into the HA MNs was determined to have optimal anti-melanogenic effects and lower cytotoxicity, as confirmed from cytotoxicity and tyrosinase inhibition studies. The GSH-loaded HA MN (GSH-HA MN) arrays were evaluated for their uniform texture, geometry, desired mechanical strength, and skin-penetrating abilities. After the application of GSH-HA MN patches to animal skin tissue, their dissolving abilities and drug-releasing patterns were studied.

## 2. Materials and Methods

### 2.1. Materials

Reduced glutathione (GSH), gelatin (from porcine skin), 3-(4,5-dimethylthiazol-2-*yl*)-2,5-diphenyltetrazolium bromide (MTT), and kojic acid (KA) was purchased from Sigma-Aldrich (St. Louis, MO, USA), sodium hyaluronate (100 k, HA) was purchased from SNVIA (Busan, Korea). Chondroitin sulfate (CS) was purchased from Wako Chemicals (Osaka, Japan). α-Melanocyte stimulating hormone (α-MSH) was purchased from TOCRIS (Bristol, UK), and apoptosis detection kit using FITC (fluorescein isothiocyanate)-labeled annexin V was purchased from BD Biosciences (Bedford, MA, USA). A liquid prepolymer (Sylgard 184A) and a curing agent (Sylgard 184B) for the polydimethylsiloxane (PDMS) molding were purchased from Dow Corning (Midland, MI, USA). All chemicals were used without further purification. Porcine skin (hair removed) was procured from a local butcher shop and kept at −20 °C until it was used in experiments.

### 2.2. Cell Culture

Human keratinocytes (HaCaT cells) and mouse-derived melanoma cell lines (B16F10), which were used for in vitro studies, were obtained from ATCC (Manassas, VA, USA). Cells were grown in GSH-free DMEM medium (Hyclone, Logan, UT, USA), supplemented with 10% fetal bovine serum (FBS, Hyclone), 2 mM glutamine (Sigma-Aldrich), 100 U/mL penicillin (Hyclone), and 100 ug/mL streptomycin (GenDEPOT, Barker, TX, USA) at 37 °C in a humidified atmosphere with 5% CO_2_.

### 2.3. Odor Tests

The smell scoring method was performed to evaluate the reducing effect of biopolymers on the GSH odor [4]. The quantity of biopolymer (sodium hyaluronate, gelatin, and chondroitin sulfate) was fixed at 10% by weight in the total solution and GSH was mixed at different concentrations (1.0%, 2.0%, 2.5%, and 5.0% by weight) in deionized (Di) water. After 30 min of mixing, a test panel of 10 volunteers smelled a solution containing a mixture of biopolymer and GSH. The GSH solution without a biopolymer was used as a reference to compare the sulfurous odor of the mixed samples. Each volunteer was asked to indicate their perception of the mixed solution’s odor using the following 5-point scale: 1: no odor, 2: recognizable odor, 3: easily noticeable odor, 4: strong odor, and 5: intense odor.

### 2.4. Gas Chromatography (GC) Measurements

Based on scoring results, the amount of released H_2_S was quantified for all GSH-HA formulations by gas chromatography (Shimadzu, Tokyo, Japan) using a Pulsed Frame Photometric Detector (PFPD) [23,24]. Solutions of 1%, 2.5%, and 5% (by weight) GSH was mixed with 10% (by weight) of HA, and 1 mL was injected into a 10 mL brown vacuum vial. The vial was stored in an oven at 40 °C for 1 h and then taken out. The generated gas (100 µL) was collected using a syringe and injected into a Shimadzu GC equipped with a PFPD. The separation of the major sulfurous compounds was achieved on a 30 m × 0.25 mm i.d. glass column (DB-1, J&W) at 90 °C and a carrier flow (nitrogen) of 1 mL/min. The detection and injection temperatures were 250 and 150 °C, respectively. The quantity of H_2_S was measured using the standard samples. The sensitivity of the PFPD was 10 ppb for H_2_S. The data of all samples were confirmed to be in the range of the standard curve.

### 2.5. Cytotoxicity Tests

Cytotoxicity of GSH was assessed using an MTT assay and annexin V-FITC staining. In brief, HaCaT cells (cell number: 1 × 10^4^) were treated with 0.1, 0.25, 0.5, and 1.0 mg/mL of GSH in DMEM media. After 24, 48, and 72 h of treatment, 50 µL of MTT solution was added to the cell and incubated for 1 h before aspirating the medium. After that, 100 µL of dimethyl sulfoxide (DMSO) was added and the percentages of viable cells were calculated by measuring the absorbance of 540 nm using a MULTISKAN GO reader (Thermo Scientific, Waltham, MA, USA). Apoptosis was measured by simultaneous staining with annexin V-FITC and propidium iodide (PI). HaCaT cells (1 × 10^6^) were treated with 0.1, 0.25, 0.5, and 1.0 mg/mL of GSH. After 72 h treatment, cells were harvested, trypsinized, washed with cold PBS, and stained with 1X binding buffer containing annexin V-FITC solution and PI. Subsequently, cells were incubated at room temperature for 15 min in the dark. The stained cells were analyzed by flow cytometry within 1 h. Both apoptotic and live cells were analyzed using a Becton Dickinson FACSscan flow cytometer and BD FACSDiva software (BD Biosciences, San Jose, CA, USA).

### 2.6. Determination of Cellular Melanin Content and Tyrosinase Activity

The proposed whitening effect of GSH was evaluated by measuring its ability to inhibit tyrosinase activity. In brief, mouse-derived B16F10 melanoma cells were seed in a 6-well culture plate at a concentration of 5.0 × 10^4^ cells/well and allowed to attach overnight. Cells were treated with 0 (blank), 0.1, 0.25, 0.5, and 1.0 mg/mL of GSH and 10 µM of kojic acid (KA) as a positive control for 4 h, and subsequently stimulated with α-MSH (1 µM) for 72 h to induce tyrosinase activity. After removing media, the cells were dissolved in 500 µL of 1N NaOH and incubated for 1 h at 60 °C to solubilize melanin. The melanin content was determined by measurement of absorbance at 405 nm. For tyrosinase activity, the cells were lysed in 100 µL of 50 mM sodium phosphate buffer (pH 6.5) containing 5 µL of 1% Triton X-100 and 5 µL of 0.1 mM phenylmethylsulfonylfluoride. After centrifugation at 10,000 g (30 min, 4 °C), the supernatants with 20 µL of l-3,4-dihydroxyphenylalanine (l-DOPA) were loaded to a 96-well plate, and absorbance was measured at 492 nm at 37 °C.

### 2.7. Fabrication of GSH-MN Patches

The bullet-shaped GSH-MN arrays (10 × 10 MNs/cm^2^) were fabricated with a reusable PDMS mold by the solvent casting of aqueous solution of 10% HA solutions with different GSH concentrations (0%, 1.0%, 2.5%, and 5.0% by weight). The negative PDMS molds with bullet-shaped cavities were replicated from metal MN arrays manufactured by micromachining [16]. MN-forming solutions (350 µL of HA or HA/GSH solutions) were pipetted into the PDMS mold and dried at 40 °C for 12 h following degassing under vacuum. The dried MN arrays were gently peeled off from the mold. The morphology of the fabricated MN arrays was characterized using digital (AM413ZT, Dino-Lite, Taiwan) and optical (Eclipse TS100, Nikon, Japan) microscopy. The HA MNs containing 0, 1, 2.5, and 5 mg/mL of GSH were referred to as GSH_0_-HA MN, GSH_1_-HA MN, GSH_2.5_-HA MN, and GSH_5_-HA MN, respectively.

### 2.8. Fracture Tests

A single HA MN and GSH-loaded HA MNs (GSH_0_-HA MN, GSH_1_-HA MN, and GSH_2.5_-HA MN) were fixed using cyanoacrylate glue (Loctite 401, Loctite Corp, Dublin, Ireland) to pin the mounting stub placed on the lower grip of the universal testing machine (UTM, A&D 5000H, A&D Sales Corp, Daegu, Korea). The upper grip of the mechanical tester was moved axially down towards the MN tip at a rate of 0.1 mm/min. The yield force was measured as a function of the rate of probe displacement and expressed in Newtons (N).

### 2.9. Skin Insertion Test

The skin insertion test was performed to check the penetrating ability of MNs, the probable extent of distortion, and possible tissue deflection during its insertion. The GSH_0_-HA MN, GSH_1_-HA MN, and GSH_2.5_-HA MNs were fixed with cyanoacrylate glue to the metallic surface via attachment with the upper grip of the UTM and inserted with a force of 10 mm/min into excised porcine skin (3 × 3 cm^2^, ~2 mm thick) placed at the base of the mechanical tester [25]. The porcine skin was used after removing the subdermal fatty layer using a scalpel.

### 2.10. Dissolution Test

GSH_2.5_-HA MN was applied onto porcine skin (3 × 3 cm^2^) at several predetermined time points (3, 5, 7, and 10 min). After removing the MN patches, the dissolved height of the MN tips was measured using an optical microscope (Eclipse TS100, Nikon, Tokyo, Japan).

To confirm the punch mark of GSH_2.5_-HA MN patches, 0.5 mg of dye (Rhodamine B) was added to 1 mL of 2.5% (by weight) mixed GSH solutions to prepare the MNs. Subsequently, MN array fabrication using a solution mixed with dye was manufactured using the same method described above. The punch mark formed from the MN arrays, their position during translation into the porcine skin, and degree of tissue deflection were analyzed under a digital microscope (AM413ZT, Dino-Lite, Taiwan). Any change in the morphology of the MN following insertion was examined under an optical microscope.

### 2.11. Loading Capacity and Encapsulation Efficiency

To determine the quantity of GSH loaded into the HA MNs, the MN tips from the GSH_1_-HA MN and GSH_2.5_-HA MN respectively, were cut and fully dissolved in PBS (pH 7.4) for 24 h. The quantity of GSH amount in the MN tips was determined by analyzing 10 μL of sample solution using high-performance liquid chromatography (HPLC, e2695, Waters, USA) on a SunFire C18 column (100Å, 5 μm, 4.6 × 250 mm, Waters). The HPLC was operated using acetonitrile-water (5:95) and 0.1% trifluoroacetic acid (TFA) as a mobile phase with a flow rate of 0.8 mL/min. Based on HPLC data, the loading capacity (LC) and the encapsulation efficiency (EE) were calculated using the following equations:

(1) $$\mathrm{Loading}\;\mathrm{capacity}\;(\%)\;=\;\frac{m_{en}}{m_{tot}}\times 100$$

(2) $$\mathrm{Encapsulation}\;\mathrm{Efficiency}\;(\%)\;=\;\frac{m_{en}}{m_{in}}\times 100$$

where, *m_en_* represents the encapsulated drug amount in MN tips, *m_tot_* is the total mass of MN tips, and *m_in_* represents the drug quantity in MN-forming solutions [26].

### 2.12. In Vitro GSH Skin Permeation Tests

To investigate the ex vivo permeation kinetics across the skin of the reduced-GSH released from the GSH_2.5_-HA MN patches, static diffusion Franz cell tests were performed to calculate the rate of time-dependent GSH release through GSH_2.5_-HA MNs and its diffusion through the skin along with a reference GSH-HA solution. The GSH_2.5_-HA MN patches and 350 μL GSH_2.5_-HA solution used for MN fabrications were applied on excised Sprague-Dawley (SD) rat skin (~2 mm thick) placed between the donor and receptor chambers in the Franz diffusion cell, respectively. After inserting the GSH_2.5-_HA MN patches into the rat skin, a hydrocolloid adhesive patch (NeoDerm Roll, EVERAID, Yangsan, Korea) was applied to the MN backing during drug delivery. The receptor chamber with a side arm was filled with 22 mL of fresh PBS buffer (pH 7.4) and maintained at 37 °C [27]. One milliliter of sample was withdrawn at each time point (0.5, 1, 2, 4, 8, 12, 24, 36, and 48 h) from the Franz cell receptor chamber and refilled with an equal quantity of fresh PBS (pH 7.4). The GSH_2.5_-HA MN patches were removed from rat skin after 1 h. The quantity of GSH that was released from the MN tip and permeated through rat skin was analyzed by HPLC using the protocol described above. GSH was detected by measuring absorbance at 385 nm and the concentration of the drug was expressed in mg.

### 2.13. Statistical Analysis

All results are expressed as the mean ± standard deviation (SD) and analyzed using the Student’s *t*-test. The level of significance was set at *p* < 0.05.

## 3. Results and Discussion

### 3.1. Screening Tests to Select Deodorizable Polymers

**Figure 1:**
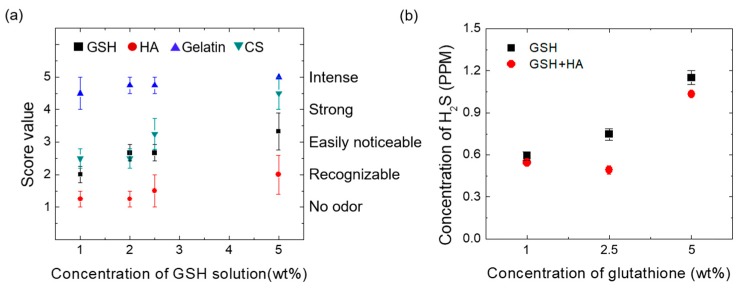
(**a**) Scoring analysis for odor test of reduced glutathione (GSH) and GSH-biopolymer formulations. Each volunteer (*n* = 10) was asked to indicate the perceived mixed solution odor intensity using the following 5-point scale: 1: no odor, 2: recognizable odor, 3: easily noticeable odor, 4: strong odor, and 5: intense odor. (**b**) Gas chromatography (GC) analysis of released H_2_S from GSH and GSH-hyaluronic acid (HA) formulations by GC-flame photometric detector.

Based on odor intensity, the scoring analysis was performed using a scale of 1–5 for evaluating the release of H_2_S from the auto-degradation of free GSH and GSH-biopolymer formulations. The experiment was performed by including 10 randomly selected healthy volunteers of either sex. A strong odor was associated with a higher quantity of released H_2_S and vice-versa. All concentrations (1.0–5.0% by weight) of gelatin (4.75 ± 0.2) and CS-GSH (3.25 ± 0.95) formulations have scored higher while HA-GSH (1.5 ± 0.35) scored lower than GSH alone (2.75 ± 0.61; Figure 1a). The high scores for the gelatin-GSH and CS-GSH formulations compared to GSH alone was due to their characteristic odors in addition to the odor of the released H_2_S. Later, based on odor scores, the quantitative estimation of released H_2_S (in ppm) was carried out by GC for the GSH-HA formulation at different concentrations. The amount of released H_2_S was found to be at a minimum for 1.0 % and 2.5 % (0.55 ± 0.01 and 0.49 ± 0.03 ppm) and at a maximum for 5.0 % (1.03 ± 0.01 ppm) of GSH in the formulation. At all concentrations, the values were found to be less than that of GSH alone (0.59 ± 0.03, 0.75 ± 0.04 and 1.15 ± 0.05 for 1.0 %, 2.5 %, and 5 % of GSH, respectively; Figure 1b). The reason for this result was that the substituted Na^+^ in HA reacts with the thiol group of GSH via the reaction formula shown in Supplementary Figure S1. It was confirmed that the reduction in odor was caused by the decreased production of H_2_S (Supplementary Figure S1) [28,29]. These results show that HA has a better deodorant effect compared to gelatin and CS, which is why it was selected for the fabrication of MNs in the present study. Since the maximum deodorizing capacity of HA for released H_2_S was observed with the 1.0 %, 2.5 %, and 5.0 % GSH-HA formulations (Figure 1b), these three concentrations were selected for fabricating MNs.

### 3.2. Effect of GSH on Cytotoxicity and Tyrosinase Activity

**Figure 2:**
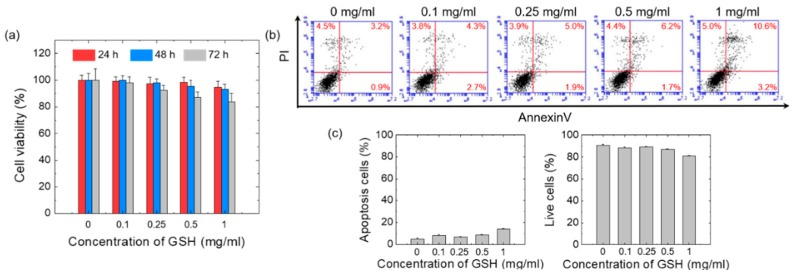
(**a**) MTT assay of GSH -treated HaCaT cells. (**b**) FACS analysis of propidium iodide (PI) uptake and annexin V binding in non-permeabilized cells (lower left, live cells; lower right, early apoptotic cells; upper right, late apoptotic cells, upper left, necrotic cells). The quantification of (**c**) apoptotic cells and live cells from three independent experiments.

The MTT assay was used to determine whether GSH is cytotoxic to HaCaT. A fluorescence activated cell sorter (FACS) analysis was performed using annexin V, which specifically and strongly binds to cell surface PI in order to confirm whether apoptotic or necrotic cell death was observed [30]. Treatment with GSH 0.2–1.0 mg/mL did not significantly change the proportion of viable cells at 72 h compared to vehicle-treated cells (Figure 2a). No cytotoxic effects of GSH on the HaCaT cells were confirmed by flow cytometry analysis using annexin V. The exposure of HaCaT cells to 0, 0.1, 0.25, 0.5, or 1 mg/mL GSH for 72 h resulted in the concentration-dependent induction of apoptosis (Figure 2b,c). Exposure to GSH did not cause a significant increase in apoptotic cells (7.90 ± 1.02–13.99 ± 0.37%) and decrease in the proportion of live cells (88.38 ± 1.02–80.81 ± 0.67%) compared to vehicle-treated cells (4.85 ± 0.72% and 90.47 ± 0.93% for apoptotic and live cells respectively; Figure 2b,c). These results suggest that GSH is not toxic to human keratinocytes and may be safely used on human skin.

**Figure 3:**
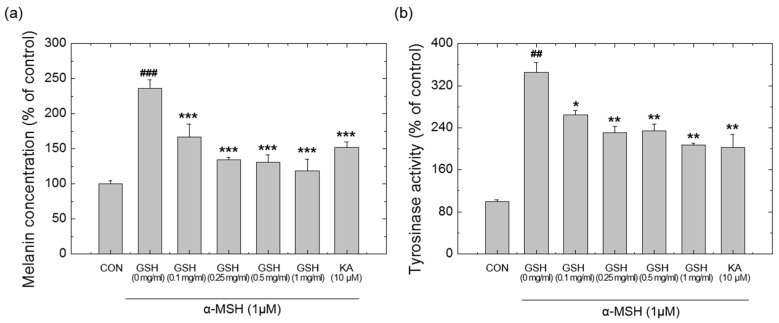
(**a**) Inhibitory effects of GSH and α-MSH on melanin production in B16F10 cells. (**b**) Inhibitory effects of GSH and α-MSH on tyrosinase activity. Kojic acid (KA) was used as a positive control. Each value represents the mean ± standard deviation (SD) from triplicate experiments. (##, ###, *p* < 0.01, 0.001, respectively. significant versus vehicle-treated; *, **, *** *p* < 0.05, 0.01, 0.001, respectively. significant versus α-MSH alone).

Tyrosinase is the enzyme responsible for melanin synthesis. The whitening effects of GSH were evaluated by measuring the inhibition rate of both melanin production and activity of tyrosinase, the rate-limiting enzyme in melanogenesis. B16F10 cells were pre-treated with 10 µM KA and GSH (0.1–1.0 mg/mL) for 4 h and then stimulated with α-MSH (1 µM) for 72 h. GSH treatment significantly inhibited melanin production induced by α-MSH (Figure 3a). In accordance with this finding, α-MSH-induced tyrosinase activity (345.60 ± 18.29% of no GSH treatment) was significantly reduced to 207.34 ± 2.78% in the treatment of 1 mg/mL GSH (Figure 3b).

### 3.3. Fabrication of GSH-loaded HA MNs and Their Mechanical Properties

**Figure 4:**
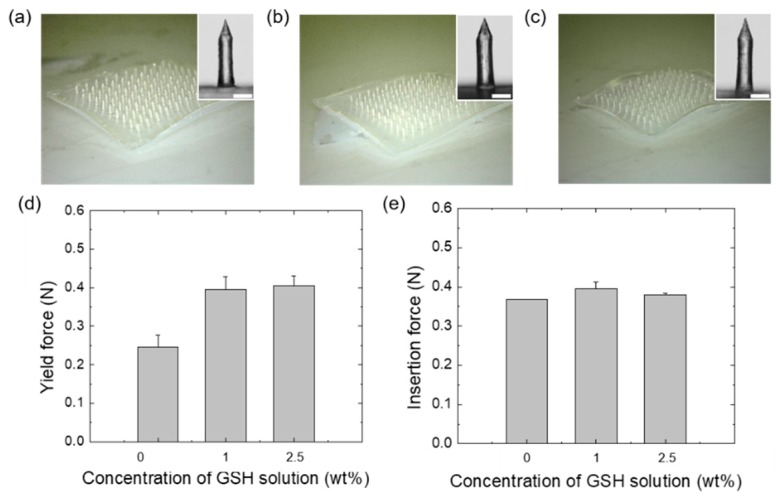
Photographs for (**a**) GSH_0_-HA MN, (**b**) GSH_1_-HA MN, and (**c**) GSH_2.5_-HA MN patches prepared from HA solutions with different GSH concentrations (0%, 1%, and 2.5%, respectively). Scale bar = 200 μm. (**d**) Yield force and (**e**) insertion force of GSH-HA MNs.

To deliver GSH across the skin in a minimally invasive manner, we selected HA as an MN material based on its capacity to reduce H_2_S that was confirmed from scoring tests and gas chromatographic data (Figure 1). The MN-forming solutions containing HA and GSH were used for the preparation of GSH-loaded HA MN (GSH-HA MN) arrays. The arrays were prepared via a solvent casting method using a PDMS mold having bullet-shaped cavities. The GSH-MN arrays (100 MNs/cm^2^) exhibited a uniform pattern with a height of 770 ± 5 µm, base diameter of 172.25 ± 2.5 µm, and a tip diameter of 7.8 ± 2.5 µm with an angle of 40 ± 2.5° (Figure 4a). The geometry of MN was determined to ensure penetration of the SC layer and transdermal delivery of GSH with minimal vascular or neuronal damage to avoid pain and capillary bleeding at the application site. The uniform features of GSH-HA MNs were obtained when the MNs were prepared using MN-forming solutions containing 2.5% GSH, but some bent MNs were observed when prepared with a high concentration (5%) of GSH (Supplementary Figure S2).

The mechanical properties of GSH-HA MNs were examined in a compression mode. A single MN affixed with a cyanoacrylate glue on the bottom surface of UTM was axially compressed by a flat metal plate attached using the upper grip of the machine at the rate of 0.1 mm/min (Figure 4a–c). The yield force, or the force required for a material to lose its elasticity, was determined to be 0.25, 0.39, and 0.40 N with a deviation of ±0.03 for GSH_0_-HA MNs, GSH_1_-HA MNs and GSH_2.5_-HA MNs, respectively (Figure 4d). The insertion force was found to be 0.36 ± 0.003, 0.39 ± 0.015, and 0.37 ± 0.005 for GSH_0_-HA MNs, GSH_1_-HA MNs and GSH_2.5_-HA MNs, respectively. Both the yield force and insertion force were found to be enough for the insertion of MNs into porcine skin without any fracture, distortion, or tissue deflection.

### 3.4. In Vitro Dissolution Tests of the GSH-HA MNs

**Figure 5:**
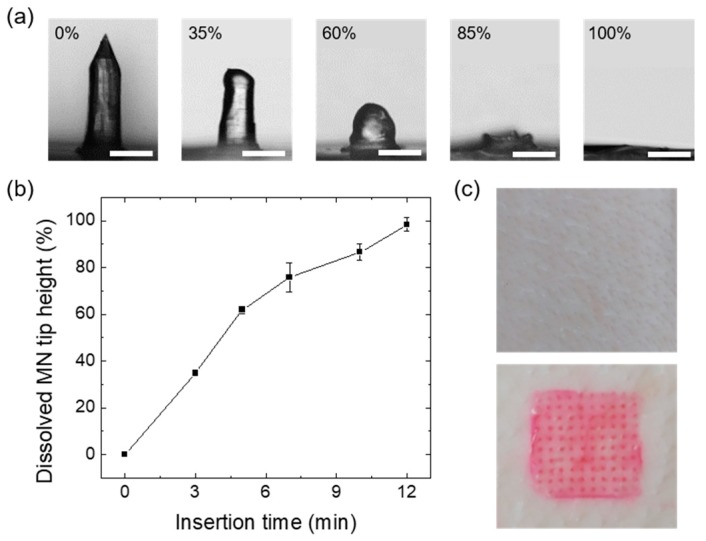
(**a**) Optical microscopic images of GSH-MN taken before (0%) and after partial dissolution (35%, 60%, 85%, and 100%) of the MN tip following application into porcine skin (scale bar = 200 μm). (**b**) Plot for the dissolved tip height as a function of insertion time. (**c**) Photographic images showing the porcine skin before (top) and after (bottom) application of the dye-loaded GSH-MN patches.

The dissolution time of a biopolymer determines the pattern of drug release and the onset of action for any formulation. To predict the time required for the release of GSH, a single MN (GSH_2.5_-HA) was inserted into porcine skin, which is structurally similar to human skin. Microscopic analysis showed the time-dependent dissolution of the MNs. After applying GSH-MNs to the skin for 12 min, MN tips were completely dissolved, which initiated its pattern of rapid release (Figure 5a,b). To confirm skin penetration of the GSH_2.5_-HA MN patches, MN patches (1.5 × 1.5 cm) loaded with rhodamine dye were applied to a porcine skin with an insertion force of 20 N for 1 min. After removing the dye-loaded MN patches, we observed an array of dye spots corresponding to each needle, confirming the insertion of the GSH_2.5_-HA MNs into the skin (Figure 5c).

The loading capacity refers to the maximum amount of drug that can be subjected to a delivery carrier and determines its loading efficiency. The loading capacity of GSH-HA MN patches is defined as the mass of the loaded drug (GSH) in MN tips divided by the total mass of GSH-HA MN tips. To check the loading efficiency, GSH-loaded MN tips were cut and dissolved in PBS (pH 7.4), and the amount of GSH loaded into the tips was analyzed by HPLC. The loading amount of GSH in 100 MN tips was 0.29 ± 0.03 mg and 0.56 ± 0.03 mg for GSH_1_-HA MN patches and GSH_2.5_-HA MN patches, respectively. Given the total mass of GSH-MN tips (100 MNs, 2 ± 0.2 mg), the loading capacity was 11.26 ± 1.24% and 21.33 ± 1.13% while the encapsulation efficiency was found to be 8.36 ± 0.92% and 6.34 ± 0.34% for GSH_1_-HA MN and GSH_2.5_-HA MNs, respectively (Table 1). Similar values between the HA:GSH weight ratio (10:1 or 10:2.5) in MN-forming solutions and the loading capacity of GSH in MN patches suggest the uniform distribution of GSH in the MN patch.

### 3.5. Ex Vivo Skin Permeation Test of GSH-HA MN Patches

**Figure 6:**
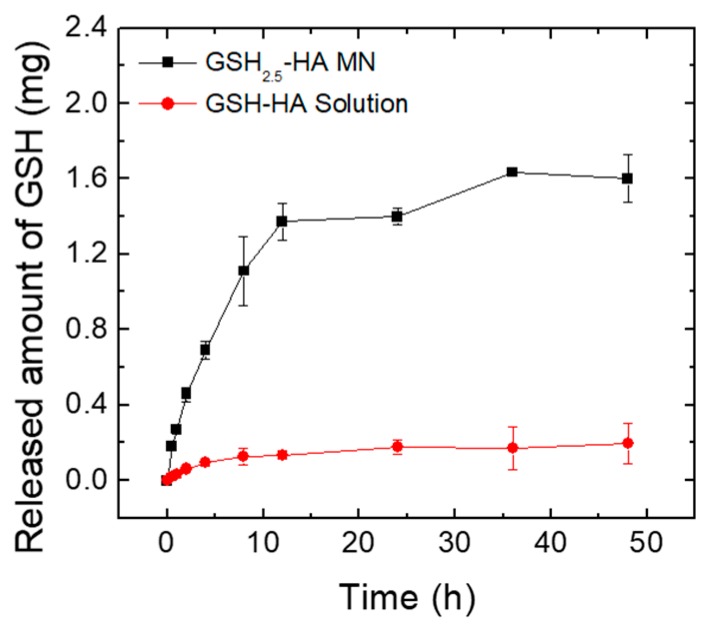
In vitro release profiles of GSH from GSH_2.5_-HA MN patches and GSH_2.5_-HA solution analyzed by the Franz cell assay.

To estimate the onset as well as the duration of GSH release from GSH_2.5_-HA MNs, the Franz cell study was performed for 48 h by placing the skin of a SD rat under simulated physiological conditions (Figure 6). The GSH_2.5_-HA MNs showed relatively fast linear release kinetics for the initial 12 h followed by a slow rate of release over time.

The permeated GSH amount across skin after MN application was ~1.4 mg for the initial 12 h of the experiment and reached to ~1.6 mg for 48 h. A quantity of GSH greater than the loaded amount in MN tips was delivered. This amount not only includes the GSH loaded in the GSH_2.5_-HA MN tips but also the GSH incorporated in the base layer as a drug reservoir. In contrast, the GSH-HA solution was slowly delivered in small amounts (~0.2 mg) due to poor skin permeation of GSH. As transdermal delivery of human growth hormone using MNs prepared by carboxymethylcellulose and trehalose demonstrated a short t_max_ (~30 min) after MN application and exhibited similar pharmacokinetic profiles with that of the SC injection [19], the GSH-HA patch with fast water-absorption properties would be applicable for systemic GSH delivery. Based on in vitro study, the treatment of 1 mg/mL GSH significantly inhibited melanin production and tyrosinase activity. Considering the small volume (~150 µL/cm^2^) of interstitial fluid in skin [31], locally delivered GSH by HA MN patches (1.6 mg GSH/1 cm^2^ MN patch) could act as an antioxidant agent during the MN dissolution process. Since the release kinetics of GSH loaded in MNs can be controlled by the crosslinking density of the MN material [32,33], a swellable MN platform would be beneficial for effective GSH delivery with minimal side effects.

## 4. Conclusions

Based on the deodorizing capacity (score value) and results of GC, we selected HA to develop biodegradable MN patches for odorless transdermal delivery of GSH. HA masked the foul odor of GSH more efficiently than gelatin and CS. The HA-MNs patch (GSH_2.5_-HA) had enough durability to penetrate through the skin without being damaged. The release of GSH from the HA-MNs patches (GSH_1_-HA and GSH_2.5_-HA) was found to be uniform over a given time period, as shown by respective experiments. The cytotoxic and tyrosinase inhibitory studies showed GSH concentration of 1 mg/mL to be safe and effective for skin whitening purposes. Altogether, the feasibility of the developed HA MN patches as a promising platform for transdermal delivery of GSH or drugs with noxious or unacceptable organoleptic properties has been demonstrated based on experimental data claimed in this study.

## Figures and Tables

**Table 1:** Amount of GSH loaded in GSH-HA MN patches.

GSH-Loaded MN Patch	GSH Amount Loaded in 100 MN Tips (mg)	Loading Capacity of GSH (%)	Encapsulation Efficiency of GSH (%)
GSH_1_-HA MN	0.29 ± 0.03	11.26 ± 1.24	8.36 ± 0.92
GSH_2.5_-HA MN	0.56 ± 0.03	21.33 ± 1.13	6.34 ± 0.34

## Supplementary Materials

The following are available online at https://www.mdpi.com/1999-4923/12/2/100/s1, Figure S1: The effect of sodium hyaluronate on odor reduction, Figure S2: GSH_5_-HA MN array images.
