# Supplementary material for: Odorless Glutathione Microneedle Patches for Skin Whitening
Source: Pharmaceutics. 2020 Jan 27;12(2):100. doi: 10.3390/pharmaceutics12020100 (PMC7076458; doi:10.3390/pharmaceutics12020100)
Supplement: Supplementary file 1 [file pharmaceutics-12-00100-s001.pdf]

Supplementary Materials: Odorless Glutathione Microneedle Patches for Skin Whitening

Yechan Lee, Sujeet Kumar, Sou Hyun Kim, Keum-Yong Seong, Hyeseon Lee, Chaerin Kim, Young-Suk Jung and Seung Yun Yang

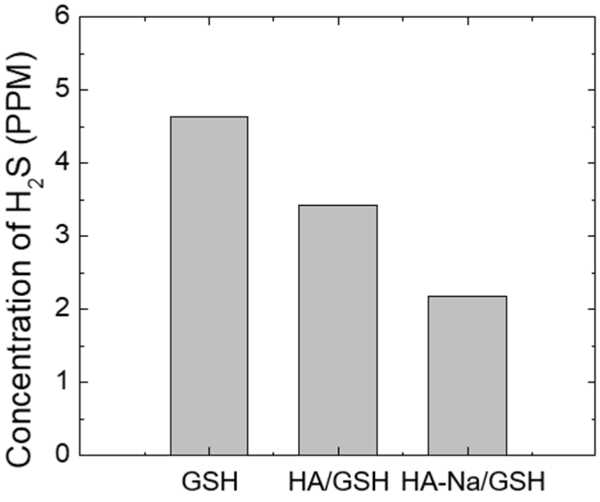

Figure S1. The odor-reducing effect of HAs through GC analysis results in released H_2S from GSH (2.5%), hyaluronic acid (HA/$GSH_{2.5}$) and sodium hyaluronate (HA-Na/$GSH_{2.5}$) formulations.

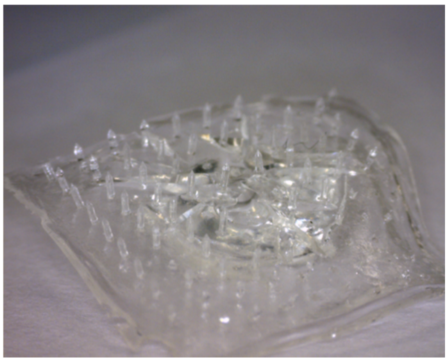

Figure S2. Photographic image of the GSH_5-HA MN array.
